# The Link between Oral and General Health

**DOI:** 10.1155/2019/7862923

**Published:** 2019-05-29

**Authors:** Wael Sabbah, Morenike Oluwatoyin Folayan, Maha El Tantawi

**Affiliations:** ^1^King's College London, Faculty of Dentistry, Oral & Craniofacial Sciences, Denmark Hill, London SE5 9RW, UK; ^2^Department of Child Dental Health, Obafemi Awolowo University, Ile-Ife, Nigeria; ^3^Preventive Dental Sciences Department, College of Dentistry, Imam Abdulrahman Bin Faisal University, P.O. Box 1982, Dammam 31441, Saudi Arabia

The relationship between oral health and general health has been the focus of research interests for decades. While the impact and oral manifestations of certain systemic conditions have been identified very early [1, 2], later research examined the potential impact of oral diseases on chronic systemic conditions. To list a few, periodontal diseases have been linked to cardiovascular diseases, high blood pressure, stroke, diabetes, dementia, respiratory diseases, and mortality, where an inflammatory pathway was depicted [3, 4]. Another line of research examined the association between the number of teeth, severe dental caries, and general health among older adults [5, 6] and children [7], suggesting a nutritional pathway. While a causal relationship between oral health and general health is still unconfirmed, comorbidities due to common risk factors appear to be a more acceptable explanation in view of the current evidence [8]. This highlights the importance of integrating oral health and general health policies and health-promoting interventions and the importance of considering oral health status among individuals with compromised medical conditions.

In this special issue, ten articles pertaining to the oral health/general health relationship and care for medically compromised patients have been published.

J. Kowar et al. compared mortality rates among 3,902 Swedish patients who received dental implants in the edentulous arch to the national death register. The authors concluded that while the edentulous patients younger than 60 years were at a higher risk of mortality from cardiovascular diseases than the general population, those over 79 years who had implant were at a significantly lower risk of all-cause mortality than the general population.

A few of the articles examined the relationship between periodontal diseases and general health. P. B. Linhartova et al. examined 1,016 participants in a case-control study to assess the variability in the interleukin-1 (*IL-1*) gene cluster and IL-1*β* plasma levels in patients with chronic periodontitis and diabetes. Their analysis suggested that variability in the *IL-1* gene cluster may be one of the factors implicated in the relationship between chronic periodontitis and type 1 diabetes pathogenesis. Similarly, P. Naiff et al. reviewed the literatures on the relationship between chronic periodontitis and type II diabetes and concluded that periodontal screening should be part of the clinical examination of patients with diabetes. R. A. G. Khammissa et al. reviewed the literatures on the role of vitamin D serum level in maintaining periodontal health. While the authors acknowledged the limitation of the available evidence, mostly from cross-sectional studies, they acknowledged a potential role of vitamin D in maintaining good oral health.

One article examined the relationship between dental caries and maternal and child health. C. W. J. Africa and M. Turton examined oral health status and treatment needs of 443 pregnant women attending antenatal clinics in KwaZulu-Natal, South Africa. While identifying pregnant women at risk of dental caries and tooth mobility, the authors recommended early oral health screening during pregnancy to ensure wellbeing of the mother and the fetus. A. A. Alshihri et al. conducted a narrative review on the relationship between dental caries and obesity among children and adolescents. The review concluded that there is an inconsistent relationship between dental caries and body measurements. While dietary habits could explain a positive relationship between obesity and caries, the impact of severe dental pain and dental inflammation could negatively affect children's weight gain.

Others examined the oral manifestation of chronic health conditions. R. B. Chebil et al. examined 30 patients with Sjogren's syndrome to assess oral lichen planus and oral lichenoid lesions and concluded that these two lesions were common among patients with Sjogren's syndrome. F. Costantinides et al. reviewed manifestation of end-stage renal disease and hemodialysis to describe the dental operative protocols for patients awaiting kidney transplantation. The authors highlighted the need for coordination between nephrologist and dental providers and the importance of early detection of oral conditions to minimize the need for extensive care. A. Peteuil et al. examined the feasibility of the therapeutic educational program in oral health for persons with schizophrenia using the qualitative method and concluded that oral health representation evolved after the therapeutic educational program. Further study will be conducted on a larger sample to produce stronger evidence.

The manuscripts published in this special issue covered a number of different topics pertaining to the relationship between oral and general health, thus highlighting the diversity of research in this area. While few studies examined a potential relationship between oral health and mortality, diabetes, and obesity, others have addressed dental care for patients under medical care and those with medically compromised conditions. With the increasing interest in research on the oral health and general health relationship, and the high demands for providing efficient and comprehensive medical and dental care, this special issue is a timely contribution to research on this topic that will undoubtedly be beneficial to researchers working in this field of research and to oral and general health practitioners.
